# α′ formation kinetics and radiation induced segregation in neutron irradiated 14YWT nanostructured ferritic alloys

**DOI:** 10.1038/s41598-019-44508-5

**Published:** 2019-06-06

**Authors:** E. Aydogan, E. Martinez, K. March, O. El-Atwani, D. L. Krumwiede, P. Hosemann, T. Saleh, S. A. Maloy

**Affiliations:** 1 Los Alamos National Laboratory, Los Alamos, NM 87545 USA; 2 Integrated Manufacturing Center, Sabanci University, Istanbul, 34906 Turkey; 3 Eyring Materials Center, Arizona State University, Tempe, AZ 85287 USA; 4 University of California Berkeley, Berkeley, CA 94720 USA

**Keywords:** Metals and alloys, Atomic and molecular collision processes

## Abstract

Nanostructured ferritic alloys are considered as candidates for structural components in advanced nuclear reactors due to a high density of nano-oxides (NOs) and ultrafine grain sizes. However, bimodal grain size distribution results in inhomogeneous NO distribution, or vice versa. Here, we report that density of NOs in small grains (<0.5 µm) is high while there are almost no NOs inside the large grains (>2 µm) before and after irradiation. After 6 dpa neutron irradiation at 385–430 °C, α′ precipitation has been observed in these alloys; however, their size and number densities vary considerably in small and large grains. In this study, we have investigated the precipitation kinetics of α′ particles based on the sink density, using both transmission electron microscopy and kinetic Monte Carlo simulations. It has been found that in the presence of a low sink density, α′ particles form and grow faster due to the existence of a larger defect density in the matrix. On the other hand, while α′ particles form far away from the sink interface when the sink size is small, Cr starts to segregate at the sink interface with the increase in the sink size. Additionally, grain boundary characteristics are found to determine the radiation-induced segregation of Cr.

## Introduction

Nanostructured ferritic alloys (NFAs) are attractive materials for core components in Generation IV reactors due to their excellent high temperature strength, stability, and radiation damage resistance, an outcome of the existence of <5 nm nano-oxides (NOs - mostly Y_2_Ti_2_O_7_) in their microstructure^1–5^. NOs are reported to be extremely stable under both neutron and ion irradiations at the temperatures between 300 °C and 670 °C^6–12^. The interface between NOs and matrix can act as both recombination centers for point defects created from neutron damage and trapping sites for helium atoms created by transmutation reactions^1,2^.

Even though NFAs are extremely radiation resistant, radiation induced segregation (RIS) of Cr and Cr-rich alpha prime (α′) formation occur under neutron irradiation due to the high Cr content, that can affect the performance of the alloys^13,14^. There is a considerable amount of research on both ion and neutron irradiated austenitic steels indicating Cr depletion and Ni enrichment at the grain boundaries; however, the behavior of Cr is complex in ferritic steels^15–18^. Lu *et al*.^17^ investigated fifteen different ferritic and ferritic/martensitic (F/M) alloy systems and reported both enrichment and depletion of Cr at the grain boundaries under irradiation. Was *et al*.^19^ concluded that RIS behavior depends on the Cr concentration and irradiation temperatures. They found that the grain boundaries are prone to become enriched at 400 °C in T91 alloys having 9 at.% Cr while they are prone to be depleted at 500 °C in HT9 and HCM12A alloys, both having 12 at.% Cr, after 2.0 MeV proton irradiations. This has been attributed to the dominance of a vacancy mechanism at high temperatures, and an interstitial mechanism at low temperatures^20^. Similarly, Clausing *et al*.^21^ reported Cr enrichment in HT9 after neutron irradiation at 410 °C; however, the amount of segregation decreased considerably at higher temperatures. On the other hand, while investigating the irradiation temperature effects on RIS behavior of Cr in ferritic alloys, none of the above stated studies considered the influence of grain boundary type and orientation. Field *et al*.^22^ have reported that grain boundary characteristics affect the segregation behavior of Cr considerably in a 9Cr model F/M alloy neutron irradiated at 500 °C. Therefore, it is crucial to understand the relationship between RIS of Cr at grain boundaries and grain boundary character.

In ferritic alloys having a high Cr content (>8 wt.% Cr), α′ formation occurs at certain temperatures which can cause embrittlement of the materials – also known as 475 °C embrittlement^13,14,23^. The α′ formation occurs at even lower temperatures under irradiation due to local enrichment of Cr atoms as a result of displacement damage and kinetically accelerated radiation enhanced diffusion^23,24^. Mathon *et al*.^24^ reported that α′ formation occurs in Fe-Cr model alloys at the temperatures as low as 250 °C under neutron irradiation when the concentration of Cr is larger than 8 at.%. Similarly, it has been reported in Fe-Cr-Al alloys having 10–22 at.% Cr at the temperatures between 320 °C and 382 °C^25–27^. In structural alloys, such as HT9, Anderoglu *et al*.^28^ reported the formation of α′ particles up to the temperatures between 440 °C and 466 °C after neutron irradiation. α′ formation has also been observed in neutron irradiated MA957 NFAs at the temperatures between 412 °C and 430 °C and the irradiations up to 200 dpa^8,10,29^. Together with that, recently, we have reported the formation of α′ particles in 14YWT NFAs neutron irradiated at 360–370 °C up to 7 dpa^30^. It should be noted that α′ formation is closely related with the dpa rate. For instance, Pareige *et al*.^31^ have reported that while α′ formation occurs in the case of neutron irradiation (with the dose rate of ~10^−7^ dpa/sec), there is no α′ formation in the case of heavy ion irradiation (with the dose rate of ~10^−4^ dpa/sec) at 300 °C. However, it has been found that electron irradiations at 300 °C with the damage rate of 4 × 10^−5^ dpa/sec results in the formation of α′ with almost equilibrium condition^32^. Reese *et al*.^33^ observing non-equilibrium α′ concentrations as a result of heavy ion irradiations concluded that dpa rate and the ballistic mixing are the factors to effect the α′ and their concentration.

In this study, we have investigated the radiation response of a 14YWT NFA material, which was irradiated in the BOR60 reactor to 6 dpa at 385–430 °C. We report here for the first time on α′ precipitation mechanisms based on the NO distribution and grain size. It has been found that NO density decreases considerably with increasing grain size, which in turn determines the α′ size and density. Moreover, detailed microstructural analysis has been performed to understand the effect of grain boundary characteristics on the RIS behavior of Cr. It has been demonstrated that rather than the grain boundary type (i.e. low angle grain boundaries, high angle grain boundaries, coincidence site lattice boundaries), grain boundary energy poses ultimate importance to determine the Cr behavior at the grain boundaries.

## Experimental Details

### Irradiations

The nominal composition of the 14YWT NFA is 14Cr-3W-0.4Ti-0.21Y-Fe (in wt.%). The fabrication details of the plate material can be found elsewhere^34^. Cladding tubes having ~0.5 mm wall thickness were cut by electrical discharge machining (EDM) from the plate along the extrusion direction of the plate. The tubes were loaded into capsules and irradiated in BOR60 reactor (RIAR, Dimitrovgrad, Russia, 2014) for less than a year. Irradiations in the reactor were conducted at 385–430 °C to a dose of ~6 dpa with the dose rate of ~6 × 10^−7^ dpa/s.

### Pre-irradiation characterization of microstructure

Before irradiation, in order to investigate the grain size distribution, electron back scatter diffraction (EBSD) was performed in an FEI Inspect FEG scanning electron microscope equipped with a TSL EBSD detector at an acceleration voltage of 20 kV and aperture size of 50 µm. Samples were EDM cut into 3 mm disks from the tube faces and were polished flat by mechanical polishing followed by jet electropolishing using a solution of perchloric acid (5%) and methanol at −40 °C with an applied voltage of 20 V. Samples for TEM studies were prepared by standard focused ion beam (FIB) lift-out technique at 30 kV and 16 kV followed by low energy cleaning at 5 kV and 2 kV using an FEI Helios Nanolab 600 dual beam FIB instrument.

### Post-irradiation characterization of microstructure

After neutron irradiation, coupons having the size of 1.0 mm × 0.5 mm × 0.5 mm were cut with a slow speed diamond saw. Then, they were further cut into a 15 µm × 10 µm × 2 µm foils and mounted on a Cu grid at University of California Berkeley, using a standard lift-out technique with a FEI Quanta 3D FEG Focused Ion Beam (FIB) equipment. Final thinning was performed at 30 kV and 16 kV followed by cleaning at 5 kV and 2 kV at Los Alamos National Laboratory using an FEI Helios Nanolab 600 dual beam FIB instrument. High angle annular dark-field (HAADF) imaging and energy filtered transmission electron microscopy (EFTEM) were performed using an FEI Titan 80 operating at 300 kV equipped with Gatan Tridiem 863 ER/S electron energy loss spectrometer. Jump ratio images were collected at Fe-L_2,3_, Ti-L_2,3_ and Cr-M_4,5_ to visualize NOs and the α′ particles. Moreover, EFTEM was used to determine the thickness of the foils based on number of mean free paths of electrons^35^. It should be noted that the EFTEM mapping was performed at the regions having the thickness less than 50 nm in order not to have any artificial effect from foil thickness. In order to study the RIS of Cr on different grain boundaries, grain boundary characteristics were determined by Automated Crystal Orientation Mapping in TEM (ACOM-TEM)^36^. This technique enables tracking orientation changes of multiple grains simultaneously. The ACOM data acquisition was performed using a JEOL ARM200F TEM equipped with the Nanomegas ASTAR system. The data was collected scanning a <5 nm electron probe on 4 μm × 4 μm areas with a step size of 18 nm. The spot diffraction patterns were obtained using a beam precession angle of 0.4° and a camera length of 12 cm. The analyses were done only on grains with a reliability index N > 18.

In this study, 30 different grain boundaries were investigated. After determining their characteristics by ACOM-TEM analysis, RIS studies were performed on the EFTEM Cr maps. It should be noted that enrichment or depletion is determined qualitatively compared to the Cr concentration of the matrix based on the EFTEM Cr maps. Since the Cr in the matrix is removed by α′ precipitates, Cr concentration is lower compared to the unirradiated matrix composition (~14 at.% Cr). In other words, even though there is Cr enrichment at the boundaries compared to the matrix, Cr concentration might still be below 14%. Also, the measurements for denuded zone around the grain boundaries were performed at five different locations along the grain boundaries. Therefore, the error is represented by the standard deviation in the measurements.

#### Computational details

The authors have recently developed a kinetic Monte Carlo (KMC) model to study the chemical evolution of a Fe-Cr system under irradiation^37^. KMC accurately determines the microstructural evolution of the system provided that every possible event is considered with precisely calculated rates. We consider vacancy and self-interstitial hops as possible events and the rates are calculated assuming harmonic transition state theory holds. The KMC model incorporates physically accurate thermodynamic driving forces and kinetic coefficients^16,38–40^. For defect migration, the atomic interactions are based on ab-initio calculations for both the minimum and saddle point configurations. Entropic effects are also considered, leading to a model that faithfully reproduces the complex phase diagram as well as the tracer and inter-diffusion coefficients for the Fe-Cr system. This model has proven successful for studying precipitation kinetics and reproducing the dependence of Cr concentration profiles under irradiation as compared to systematic experimental studies available in the literature^20^.

Spherically ideal sinks with different sizes have been included in the simulation cell to mimic the presence of nano-oxide particles. When a defect is detected inside the sink, it is identically annihilated. This model will create a flux of defects with spherical symmetry around the particle towards the sink. The coupling of such defect flux with the flux of the alloying elements will induce enrichment or depletion of Cr near the particle. Moreover, since the Fe-14 at.% Cr alloy at 400 °C is inside the miscibility gap, the alloy presents a tendency to phase separation. To assess the effect of temperature, the system evolution has also been studied at 227 °C. Three different particle radii, namely 0.8, 1.6 and 3.2 nm, have been studied at 227 °C and 400 °C with a dose rate of 10^−6^ dpa/s, which is fairly close to the reactor irradiation conditions (~6 × 10^−7^ dpa/s).

## Results

### Microstructure before irradiation

**Figure 1:**
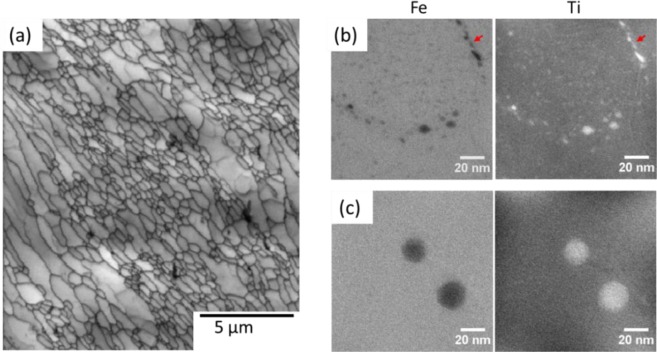
(**a**) Band contrast map showing the microstructure of 14YWT alloy before irradiation; EFTEM Fe and Ti jump ratio maps obtained from (**b**) small (<0.5 µm) and (**c**) large grains (>2 µm). Red arrows in (**b**) indicate a grain boundary.

Figure 1a shows the initial microstructure of the 14YWT tubes. It has a tri-modal grain size distribution having a grain size up to ~7 µm as reported previously^34^. Grain size can be categorized as small grains having a size less than 0.5 µm, medium size grains, between 0.5 µm and 2 µm, and large grains larger than 2 µm. Also, Fig. 1b,c show the NO distributions in small grains and large grains, respectively. One significant finding is that NO size, number density and distribution are different in large and small/medium grains. It is clearly shown that while there is a high density of NOs (<10 nm) in small grains, there are either few or no NOs inside the large grains. However, it should be noted that the particles larger than 10 nm size still exist in the large grains. NO diameter and density are measured as 2.0 ± 0.9 nm and 6.3 ± 1.2 × 10^23^ m^−3^, respectively in small grains. Moreover, NO distribution is not perfectly homogenous and NOs precipitate along some of the grain boundaries as shown with a red arrow in Fig. 1b. Similar behavior was reported using atom probe tomography techniques^41^. It is important to note that there is no grain-size dependent distribution of NO in the small and medium size grains.

### Microstructure after irradiation

**Figure 2:**
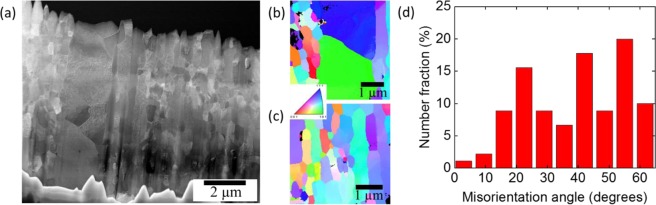
(**a)** HAADF image of the FIB foil showing large and small grains; inverse pole figure maps of (**b**) large-grained region and (**c**) small-grained region obtained by automated crystal orientation mapping technique; (**d**) misorientation angle distribution in the FIB foil in (**a**).

Figure 2a shows a HAADF image of a TEM foil taken from a 14YWT tube neutron irradiated to 6 dpa at 385–430 °C. Similar to the unirradiated condition, it consists of a multi-modal grain size distribution. It should be noted that after less than a year of irradiations up to 430 °C, there is no considerable change in the grain size distribution (~0.5 µm average grain size). Figure 2b,c show orientation maps at large grained and small grained regions, respectively. Neutron diffraction analysis has shown that the bulk texture is mostly along <100> ǁND (normal direction) and <110> ǁRD (rolling direction)^34^. On the other hand, 4 µm × 4 µm orientation maps at large and small grained regions do not show any specific texture as seen in Fig. 2b,c. Figure 2d shows the misorientation angle distribution in the FIB foil shown in Fig. 2a. Boundaries have been found to be mostly high angle grain boundaries having a misorientation angle larger than 15° (~90%). It should be also noted that the fraction of coincidence site lattice (CSL) boundaries is quite low, even lower than 5%.

**Figure 3:**
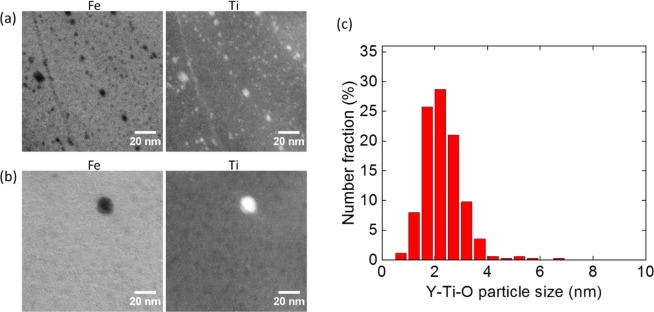
EFTEM Fe and Ti jump ratio maps obtained from (**a**) a small grain (<0.5 µm) and (**b**) a large grain (>2 µm), and (**c**) NO size distribution in small grains after 6 dpa neutron irradiation at 385–430 °C.

Figure 3a,b show the NO distribution in small and large grains after neutron irradiation. Similar to the unirradiated conditions, it is clearly seen that NOs exist in a high density in small grains while there are a few larger oxide particles in the large grains. Diameter and number density of the NOs are measured as 2.25 ± 0.93 nm and 7.0 ± 3.5 × 10^23^ m^−3^, respectively. It should be noted that measurements were performed on at least 5 small grains and statistics involve counting of ~720 NOs, in Fig. 3c. Moreover, there is no considerable change in NO size, number density and distribution after 6 dpa neutron irradiation at 385–430 °C, which is similar to the ion irradiations on these alloys^41^. On the other hand, size distribution of oxide particles in large grains is not provided due to poor statistics. Only 9 oxide particles were captured on 10 different maps on 3 large grains. Their size is mostly larger than 10 nm.

**Figure 4:**
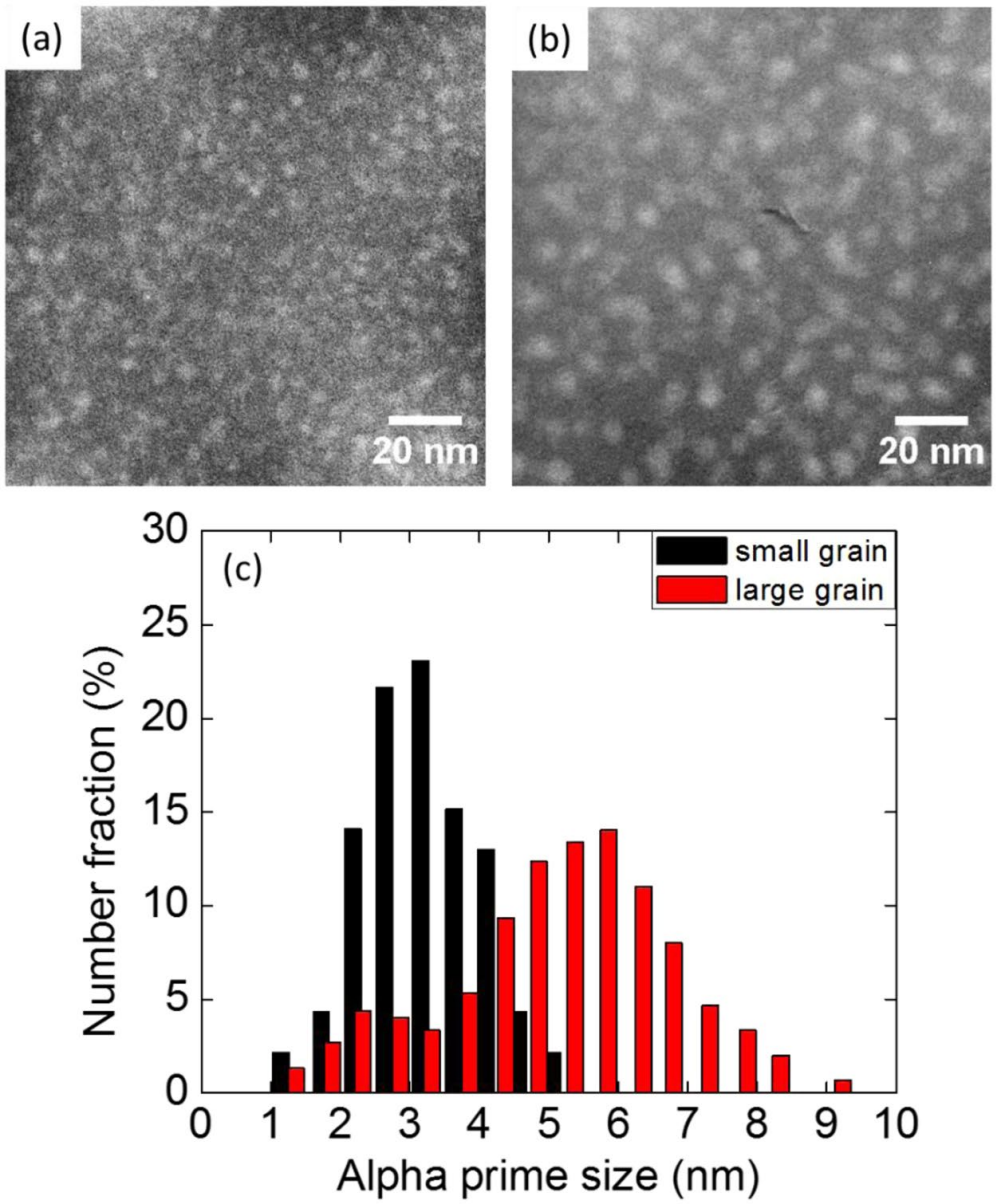
EFTEM Cr jump ratio maps showing α′ distribution in a (**a**) small and (**b**) large grain, and (**c**) α′ size distribution in these grains.

Another significant finding is that α′ size and number density are different in large and small grains. Figure 4 shows the EFTEM Cr jump ratio maps to visualize α′ distribution in small and large grains of 14YWT tube neutron irradiated to 6 dpa at 385–430 °C. Even though α′ distribution is quite homogenous in both grains, α′ size is larger and number density is lower in the large grain compared to the small grain, as shown in Fig. 4a,b. In Fig. 4c, while α′ distribution shows a Gaussian distribution in the small grain, it shows a bimodal size distribution in the large grain. Average size in diameter and number density are measured as 3.21 ± 0.84 nm and 5.97 ± 0.59 × 10^23^ m^−3^, respectively, in small grains while they are measured as 5.12 ± 1.60 nm and 1.77 ± 0.18 × 10^23^ m^−3^ in large grains.

**Figure 5:**
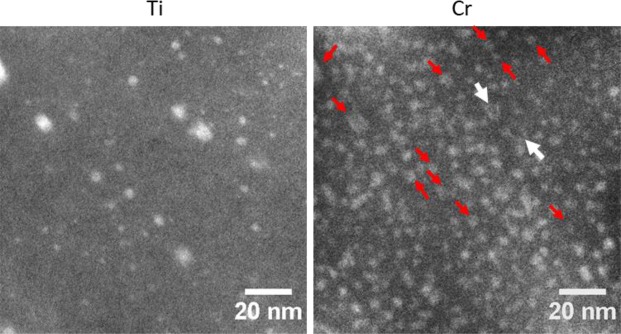
EFTEM Ti and Cr jump ratio maps to visualize NO and α′ distribution, respectively in a grain having the size ~0.5 µm. Red arrows point some of the Cr rich NOs. White arrows point two large particles having a Cr rich shell. For interpretation of the references to color in this figure, the reader is referred to the web version of this article.

**Figure 6:**
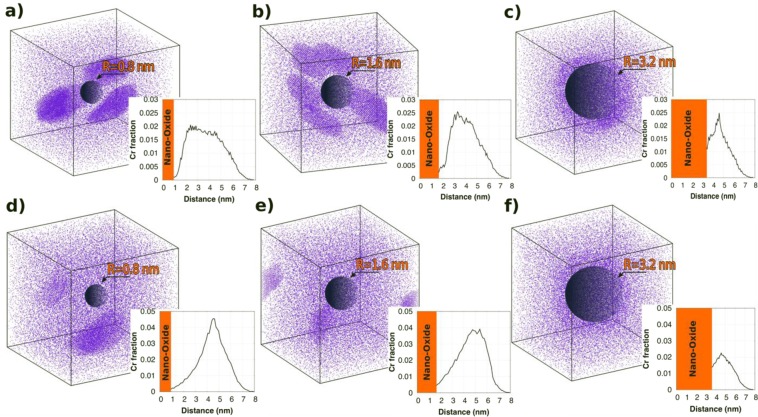
Cr distribution as given by the KMC model with a dose rate of 10^−6^ dpa/s for a spherical sink with (**a**) r = 0.8 nm, (**b**) r = 1.6 nm and (**c**) r = 3.2 nm at T = 227 °C; (**d**) r = 0.8 nm, (**e**) r = 1.6 nm and (**f**) r = 3.2 nm at T = 400 °C. The insets show the Cr radial distribution (Cr atoms/total number of Cr atoms) from the center of the spherical sink.

Figure 5 shows the Ti and Cr jump ratio maps to visualize the distribution of both NOs and α′ particles. There seems to be an anti-correlation between NOs and α′ particles. In other words, α′ particles precipitate between the NOs, rather than precipitating on them, as reported elsewhere^10,30^. It should be noted that NOs are rich in Cr, as pointed by the arrows in Fig. 5. Even though it is not clear for small NOs, large NOs pointed by white arrows indicate a Cr-rich outer rim. In order to understand segregation/precipitation behavior of Cr in the case of spherical sinks having different sizes, KMC simulations have been performed at 227 °C and 400 °C with sink radius of 0.8, 1.6 and 3.2 nm. Figure 6 shows the results from these simulations, where the Cr distribution is described. The insets in the figure present the radial Cr fraction from the center of the spherical sink. At low temperatures and for the smallest sink radius (0.8 nm, Fig. 6a), precipitates form far from the sink, where the concentration of defects is large due to the larger effective diffusion coefficient (radiation-enhanced diffusion), which leads to faster precipitation. At the same time, radiation-induced segregation tends to enrich the defect sink in Cr, which increases the supersaturation of Cr around the sinks^20^. This driving force leads to the nucleation of more stable precipitates at a later time closer to the interface, which results in the dissolution of the early-formed precipitates far from the nanoparticle (see Video I in supplemental material). The presence of the precipitates close to the sink hinders the Cr enrichment of the matrix-sink interface since Cr atoms preferentially are located in α′ precipitates nearby the sink. On the other hand, with the increase in the sink size, Cr starts to segregate at the interface. The difference between the behavior of small and large sinks (Fig. 6b,c) can be attributed to their surface area. In the case of large sinks, there are less defects in the matrix due to their larger sink strength. Thus, the driving force for Cr precipitation is lower even at regions far from the sinks. It is even more difficult for precipitates to segregate towards the interface, leading to Cr precipitation far from the sinks. At higher temperature, 400 °C, the behavior is overall similar (Fig. 6d–f). For the smallest sinks, the precipitates are formed far from the interface, as seen in the case of low temperature. However, they tend to stay far away from the sinks due to the reduced thermodynamic driving force as a result of a weaker supersaturation of Cr at this temperature. Therefore, precipitation occurs only where the concentration of defects is larger, i.e. far from the sink.

**Figure 7:**
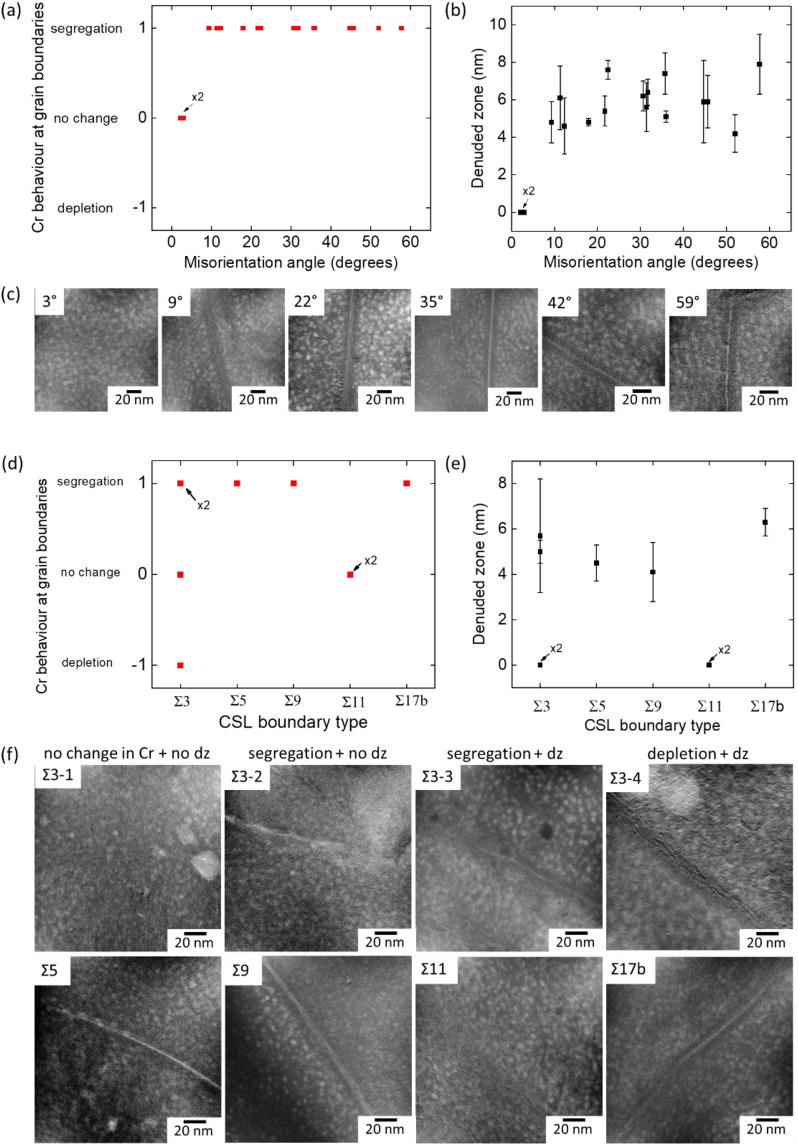
(**a**) Cr behavior at grain the boundaries and (**b**) α′ denuded zone size with respect to misorientation angle of the grain boundaries (**c**) EFTEM Cr jump ratio maps for low angle boundaries and high angle random boundaries; (**d**) Cr behavior at the grain boundaries and (**e**) α′ denuded zone size with respect to misorientation angle of the grain boundaries (**f**) EFTEM Cr jump ratio maps for coincidence site lattice (CSL) boundaries. x2 in (a), (b), (d) and (e) indicates two data points overlapping.

Figure 7 shows the Cr behavior at the grain boundaries and denuded zone size with respect to misorientation angle for low angle and high angle random boundaries (Fig. 7a,b) and the coincidence site lattice (CSL) boundaries (Fig. 7d,e). Moreover, Fig. 7c,f show the Cr maps of some of the random and CSL boundaries, respectively. It should be noted that among the thirty investigated boundaries, two of them were low angle grain boundaries having misorientation angles less than ~8°. According to Fig. 7a,b, there is no change in the Cr concentration in the case of those low angle grain boundaries having misorientation angles less than ~8° while Cr segregates at the random grain boundaries having misorientation angle larger than ~8°. Moreover, the denuded zone size varies between 0 and ~10 nm for all grain boundary types. While low angle grain boundaries having misorientation angles less than ~8° show no α′ denuded zone, random grain boundaries having misorientation angles more than ~8° show α′ denuded zone. However, it is quite difficult to conclude a trend in the denuded zone size with increasing misorientation angle. In the case of CSL boundaries, some of the Σ3 boundaries besides Σ5, Σ9 and Σ17b boundaries show α′ denuded zone. On the other hand, two out of four investigated Σ3 CSL boundaries and both of the Σ11 CSL boundaries have no change in Cr concentration and no α′ denuded zone. These findings are similar to the ones we have reported for slightly higher dose and lower temperature irradiation conditions^30^.

## Discussion

There is an anti-correlation between NOs and α′ particles as α′ particles precipitate between the NOs, seen in Figs 5 and 6. Bailey *et al*.^10^ proposed that NOs cannot provide enough sink strength to annihilate all the defects in the sample and since the defect concentration between NOs is maximum, Cr segregation and therefore α′ precipitation occurs between NOs. Similarly, KMC simulations show that α′ precipitation occurs always far from the spherical sinks. Depending on the sink strength (size of the spherical sink) and temperature, α′ particles either re-precipitate close to the interface without Cr segregation at the interface, or stay far from the interface with Cr enrichment at the interface, inferring that there is no α′ formation on the sink. However, it is worth noting that the overall kinetics in the Fe-Cr system is slow, implying that precipitate coalescence requires a fair amount of time (dose). That is why precipitates are observed in most cases far from the interface, where they nucleate due to the larger concentration of defects leading to enhanced diffusivities. However, as we described in the case of small sink radii and low temperature, at the steady state solution there is no reason for the precipitates to remain far from the interface and after an infinite amount of time, precipitates will probably aggregate close to the sink, unless ballistic mixing re-dissolves the precipitates. It should also be noted that radiation damage, represented by hardening, saturates at the damage level of 5–10 dpa^42^. Therefore, the volume fraction of α′ can be proposed to saturate below 10 dpa. However, this proposition requires further evidence at higher dose irradiations. Furthermore, it has been found that either NOs or their outer rims are rich in Cr as pointed by red and white arrows in Fig. 5. In the literature, it is reported that there is generally Cr enrichment at the sinks in Fe-Cr systems^43–45^. Cr/Ti rich shell structures in Y-Ti-O particles have been reported by many other authors before irradiation^46–48^. It has been also shown that this core-shell structure becomes more obvious with the increase in NO size^47,48^. However, detailed atom probe tomography analysis on 14YWT alloys before irradiation was not conclusive about the core/shell structure in NOs that contain Cr^41^. Thus, even though EFTEM maps in Fig. 5 infer the existence of core/shell structure, especially for the large particles, after neutron irradiation in the present conditions, it is highly possible that this structure is a result of the combination of pre-irradiation conditions and irradiation effects. This has been confirmed by the KMC simulations in Fig. 6 showing that irradiation at 400 °C induces slight Cr enrichment at the outer rim of the NO particles, especially at large sizes.

**Figure 8:**
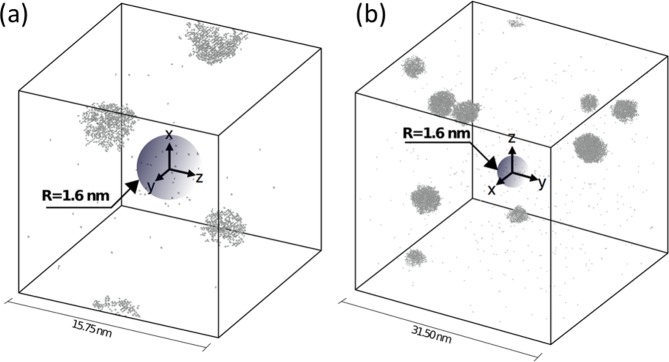
Cr precipitates forming far from a defect sink of 1.6 nm radius for a cubic system at 400 °C and 10^−6^ dpa/s with (**a**) L = 15.75 nm and (**b**) L = 31.50 nm.

Oxide dispersion strengthened (ODS) alloys have been reported to have bimodal grain size distribution due to the heterogeneous distribution of stored energy as a result of initial mechanical alloying, inducing heterogeneous recrystallization throughout the microstructure^49^. This heterogeneous microstructure is quite stable even at high temperatures and cannot be fixed by complete recrystallization^50–52^. Lu *et al*.^53,54^ have reported that NO distribution in fine and coarse grains of spark plasma sintered 9Cr ODS alloys having bimodal grain size distribution varies considerably, similar to the present study (see Figs 1 and 3). On the other hand, this is the first study reporting the α′ formation kinetics based on the NO distribution in ODS alloys, to the best of authors’ knowledge. Figure 4 shows that α′ precipitate size is smaller and density is larger in small grains compared to the large grains having larger α′ precipitates with a lower density. In order to understand the mechanism of α′ evolution based on the NO density, the KMC model was used. Figure 8 shows α′ precipitates forming far from a spherical defect sink of 1.6 nm in radius at 400 °C under a dose rate of 10^−6^ dpa/s. Two different system volumes have been tested, 15.75^3^ nm^3^ (Fig. 8a) and 31.50^3^ nm^3^ (Fig. 8b), which correspond to a volume fraction of NOs of 0.0044 and 0.00055, respectively. In both cases precipitates are formed far from the sink. In the lower volume fraction case, the concentration of defects further from the sink is larger than for the higher volume fraction case, which accounts for faster kinetics, leading to sooner nucleation of α′ precipitates and a faster growth. This compares satisfactorily with experiments, where in larger grains with lower density of NOs, larger precipitates are observed compared to smaller grains with greater NO densities with smaller α′ precipitates.

It is critical to understand the overall radiation resistance of 14YWT alloys that are considered as one of the most promising materials for Gen IV nuclear reactor applications, which are being designed for operating to hundreds of dpa. 14YWT alloys have been shown to be extremely swelling resistant^3^; however, their relatively high Cr content might lead brittleness due to the formation of α′ precipitates. Up to now, bimodal grain size distribution in mechanically alloyed NFAs has been well known; however, the inhomogeneous distribution of NOs and its effect on precipitation of α′ particles have been unclear. Therefore, this study serves as a baseline to determine the effect of grain size and NO distribution on the α′ formation in nanostructured ferritic alloys having multi-modal grain size distribution. In our previous study^30^, α′ formation in 14YWT alloys neutron irradiated to 7 dpa at 360–370 °C and other high Cr alloys irradiated at similar conditions were compared and it was found that the number density of α′ particles in 14YWT alloy is considerably low compared to the other materials. Similarly, this study clearly indicates the effect of high number density of NOs within a single material. While the volume fraction of α′ is low in small grains, it is high in large grains, resulting in an increase in the material’s brittleness. However, it should be noted that the fraction of the large grains is less than 5% of the microstructure.

**Figure 9:**
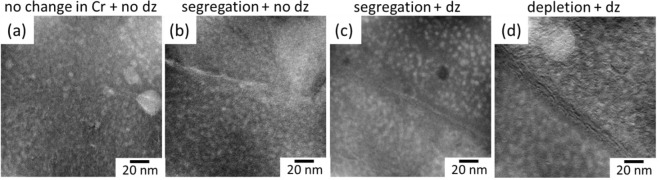
EFTEM Cr jump ratio maps showing different behavior of Σ3 grain boundaries: (**a**) no change in Cr concentration and no α′ denuded zone (**b**) segregation of Cr and no α′ denuded zone (**c**) segregation of Cr and α′ denuded zone (**d**) depletion of Cr and α′ denuded zone. (dz: denuded zone).

Grain boundary energy determines the segregation behavior of Cr at the grain boundaries. Zhou *et al*.^44^ calculated the Gibbsian interfacial excess of the boundaries in Fe-Cr nanocrystalline materials and found that the high angle random boundaries have the highest energy with highest solute concentration. Even though the energy of the CSL boundaries are lower than the HAGBs, it has been reported that the energy of the Σ3 type boundaries can vary extensively in both FCC and BCC metals based on their coherency^55,56^. In fact, segregation tendency is higher in incoherent Σ3 grain boundaries, even slightly lower than the HAGBs^44^. In the literature, segregation tendency at Σ3 boundaries has also been reported by other authors^43,45^. Besides having higher energies compared to the coherent Σ3 boundaries, incoherent Σ3 boundaries were found to be more sensitive to the inclination angle of the boundaries compared to the other CSL boundaries^44,56^. In this study, after neutron irradiation to 6 dpa at 385–430 °C, Σ3 type boundaries have been found to show extensively different behavior, in agreement with the above stated studies. Some Σ3 boundaries show neither Cr segregation nor α′ denuded zone (Fig. 9a) while some show segregation without any α′ denuded zone (Fig. 9b) besides showing either segregation or depletion with α′ denuded zone (Fig. 9c,d).

Our preliminary investigations together with the literature findings on Fe-Cr systems^43–45^ indicate that there is generally Cr enrichment at the grain boundary in the pre-irradiation condition. On the other hand, it is clear from Fig. 9 that Σ3 type boundaries behave differently after irradiation. In Fig. 9a,b, the absence of an α′ denuded zone, which is a result of low defect concentrations inhibiting the α′ precipitation^57^, infers that these Σ3 boundaries do not act as effective sinks for defects. Since the amount of defect flux is low in these boundaries, the amount of Cr flux between the matrix and grain boundary is limited. However, it should be noted that defect annihilation behavior of the grain boundaries is not linearly correlated with the Cr flux towards those boundaries^16,37^. Even though their sink behavior under irradiation is similar, the concentration of Cr at the grain boundaries infers that Σ3 boundary in Fig. 9a is coherent and the energy of the Σ3 boundary in Fig. 9b is higher compared to that in Fig. 9a. On the other hand, the existence of an α′ denuded zone in Fig. 9c,d infers that these Σ3 boundaries are effective sinks for radiation induced point defects, meaning that they are most probably incoherent. However, it is difficult to comment on the degree of initial Cr enrichment of the grain boundaries in this case. Both experimental and modelling studies have shown that Cr tends to enrich at the grain boundaries at the temperatures below ~600 °C in ferritic and F/M alloys in the absence of α′ precipitates^19,20,37,58,59^. However, Senninger *et al*.^16^ have shown that there is no considerable enrichment of Cr at the grain boundaries in 15Cr binary alloys at 377 °C. Indeed, Cr concentration was found to be lower than the nominal Cr concentration (15 at.%). In other words, in Fig. 9c, even though the grain boundary is Cr rich, it might be either enriched or depleted compared to the pre-irradiation condition. Moreover, in Fig. 9d, grain boundary is depleted in Cr after irradiation. As mentioned above, grain boundaries are mostly enriched in Cr before irradiation. Therefore, it is highly possible that irradiation induces depletion in this Σ3 boundary.

## Conclusion

In this study, we have investigated the formation kinetics of α′ precipitates and radiation induced segregation behavior of Cr in neutron irradiated 14YWT alloys (to ~6 dpa at 385–430 °C) having inhomogeneous microstructure by using detailed transmission electron microscopy techniques and kinetic Monte Carlo simulations. The main findings are listed below:

- NO size and density vary considerably in small/medium and large grains (>2 µm). In fact, there are few large oxide particles in large grains while a high number density of NOs exist in small/medium grains.
- The presence of a high number density of sinks in small grains results in smaller α′ precipitates with higher number density compared to the large grains. This has been confirmed by the KMC simulations which exhibit rapid precipitation and growth of Cr-rich particles in large grains.
- Both EFTEM and KMC results show that there is Cr segregation around the large NOs after neutron irradiation. KMC simulations at 400 °C have shown that in the presence of small sinks, α′ precipitates form far from the sink interface and there is no Cr segregation at the interface. With the increase in the sink size, Cr starts to segregate at the sink interface. At low simulation temperatures (227 °C), α′ precipitates start to form closer to the sink interface at low sink sizes. This behavior at low temperatures is attributed to higher driving force for Cr precipitation resulting in α′ precipitate formation even nearby the sinks.
- All high angle random boundaries and low angle boundaries having misorientation angles more than ~8° show segregation in Cr concentration, with α′ denuded zones of varying size.
- CSL boundaries mostly show segregation of Cr with a varying size of α′ denuded zones. However, Σ3 boundaries show different behavior (segregation or depletion or no change) which is attributed to their boundary energies depending strongly on the coherency of the boundaries.

## Supplementary information

Video 1

## Data Availability

Data supporting the findings of this study are available from the corresponding author on request.
